# PhaSeDis: A Manually Curated Database of Phase Separation–disease Associations and Corresponding Small Molecules

**DOI:** 10.1093/gpbjnl/qzaf014

**Published:** 2025-03-04

**Authors:** Taoyu Chen, Guoguo Tang, Tianhao Li, Zhining Yanghong, Chao Hou, Zezhou Du, Kaiqiang You, Liwei Ma, Tingting Li

**Affiliations:** Department of Biochemistry and Molecular Biology, School of Basic Medical Sciences, Peking University Health Science Center, Beijing 100191, China; Key Laboratory for Neuroscience, Ministry of Education / National Health Commission of China, Peking University, Beijing 100191, China; Department of Biochemistry and Molecular Biology, School of Basic Medical Sciences, Peking University Health Science Center, Beijing 100191, China; Key Laboratory for Neuroscience, Ministry of Education / National Health Commission of China, Peking University, Beijing 100191, China; Department of Biomedical Informatics, School of Basic Medical Sciences, Peking University Health Science Center, Beijing 100191, China; Department of Biochemistry and Molecular Biology, School of Basic Medical Sciences, Peking University Health Science Center, Beijing 100191, China; Key Laboratory for Neuroscience, Ministry of Education / National Health Commission of China, Peking University, Beijing 100191, China; Department of Biochemistry and Molecular Biology, School of Basic Medical Sciences, Peking University Health Science Center, Beijing 100191, China; Key Laboratory for Neuroscience, Ministry of Education / National Health Commission of China, Peking University, Beijing 100191, China; Department of Biochemistry and Molecular Biology, School of Basic Medical Sciences, Peking University Health Science Center, Beijing 100191, China; Key Laboratory for Neuroscience, Ministry of Education / National Health Commission of China, Peking University, Beijing 100191, China; Department of Biochemistry and Molecular Biology, School of Basic Medical Sciences, Peking University Health Science Center, Beijing 100191, China; Department of Biochemistry and Molecular Biology, School of Basic Medical Sciences, Peking University Health Science Center, Beijing 100191, China; Department of Biochemistry and Molecular Biology, School of Basic Medical Sciences, Peking University Health Science Center, Beijing 100191, China; Key Laboratory for Neuroscience, Ministry of Education / National Health Commission of China, Peking University, Beijing 100191, China

**Keywords:** Phase separation, Biomolecular condensation, Disease, Database, Small molecule

## Abstract

Biomacromolecules form membraneless organelles through liquid–liquid phase separation in order to regulate the efficiency of particular biochemical reactions. Dysregulation of phase separation might result in pathological condensation or sequestration of biomolecules, leading to diseases. Thus, phase separation and phase separating factors may serve as drug targets for disease treatment. Nevertheless, such associations have not yet been integrated into phase separation-related databases. Therefore, based on MloDisDB, a database for membraneless organelle factor–disease associations previously developed by our lab, we constructed PhaSeDis, the phase separation–disease association database. We increased the number of phase separation entries from 52 to 185, and supplemented the evidence provided by the original articles verifying the phase separation nature of the factors. Moreover, we included the information of interacting small molecules with low-throughput or high-throughput evidence that might serve as potential drugs for phase separation entries. PhaSeDis strives to offer comprehensive descriptions of each entry, elucidating how phase separating factors induce pathological conditions via phase separation and the mechanisms by which small molecules intervene. We believe that PhaSeDis would be very important in the application of phase separation regulation in treating related diseases. PhaSeDis is available at http://mlodis.phasep.pro.

## Graphical abstract

**Figure:**
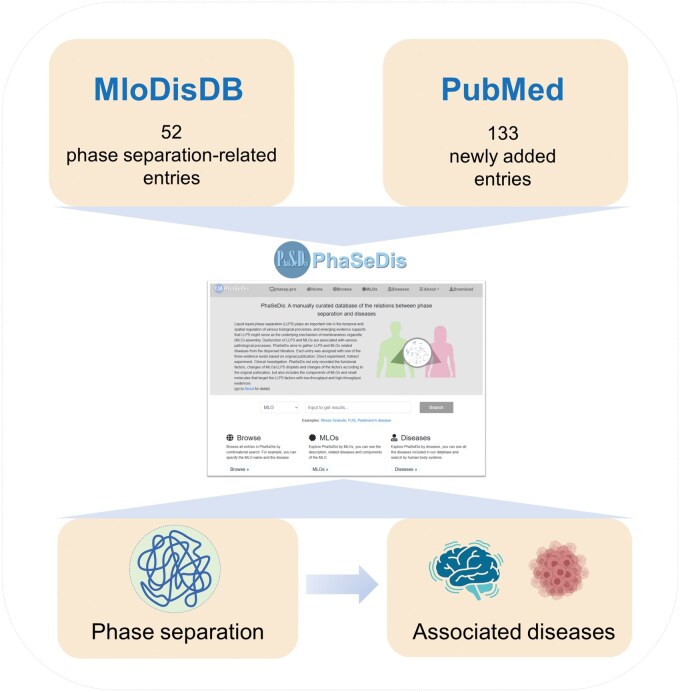

## Introduction

Phase separation (PS) is a physiochemical phenomenon where the homogeneous solution of macromolecular components separates into distinct phases, with one enriched with certain macromolecules and the other depleted of such molecules. Among many PS scenarios, liquid–liquid PS (LLPS) refers to the separation of two distinct liquid phases like oil and water, which was found to exist in a number of biological context [1]. Ever since the first observation of P granule having PS-like droplet properties, researchers in biology and medicine start to pay close attention to PS as it has the potential to unravel the forming and regulating mechanisms of membraneless organelles (MLOs) [2], such as stress granules [3], paraspeckles [4], and nucleoli [5]. Previous studies have also shown that the dysregulation of PS might be one of the vital mechanisms underlying neurodegenerative diseases or other common diseases like cancer [6,7]. Small molecules partitioned into condensates might also regulate PS, rescuing cells from a pathological state [8,9]. Therefore, understanding the role of PS in human cells, particularly in pathological context, is pivotal for the intervention of PS-related diseases and screening potential small molecules.

Researchers have been working hard in their endeavor toward investigation of biological PS [10,11]. Nevertheless, these studies are scattered, as researchers tend to carry out different experiments in verifying the condensates of interest as “PS-related bodies” [12,13]. Moreover, although there are small molecules like 1,6-hexanediol (1,6-HD) that have been proven to regulate PS [14], researchers of different backgrounds make use of a wide range of different small molecules in affecting PS and PS bodies [15–18]. Recent research indicates that small molecules can concentrate in biomolecular condensates of appropriate chemical environment, which can assist in constructing machine learning models to predict small molecule partitioning in PS bodies [19]. Many pieces of evidence have also stated that small molecules might be capable of interfering with the pathological conditions in cells since drug-applied cell lines can witness disturbance or assembly of phase separating bodies [20,21]. Thus, we believe that collecting small molecule interference examples from literature concerning valid PS-driven condensates should be of great interest to the researchers in the field.

Several databases have been established for the needs of PS researchers, including PhaSepDB [22], LLPSDB [23], PhaSePro [24], and DrLLPS [25]. While these databases highlight the properties of PS proteins and RNAs, they often ignore their functions and disease-related roles [26]. With that in mind, we established the first MLO-related disease database, MloDisDB, in 2020 [27]. Nevertheless, there have been a few limitations to MloDisDB. To begin with, the number of PS entries is limited, with only 52 entries out of the 775 MLO-related entries, which is a great neglect of PS as the possible mechanism of the formation of MLOs. Second, the annotations of small molecule interactions do not present, which is of vital significance for researchers who wish to evaluate the PS properties of different factors and screen potential drugs interacting with the PS-determining region of the protein, reversing the abnormal state of the cell. Thus, in this study, we present PhaSeDis, where we supplemented novel data and annotated the *in vivo* experimental evidence and related small molecules. PhaSeDis contains 931 entries, including 185 PS-related entries. We hope that the newly designed database would be of great assistance in the PS field.

## Database implementation

### The new database doubles the number of entries related to PS

Our previous MloDisDB was released in 2020, which is the first literature-based database of its kind that integrates the relations of MLO-related biomolecules and diseases. Nevertheless, of all the 775 entries upon the first release, only 52 entries are annotated as PS-related biomolecules, while the rest are correlated with certain MLOs. These years have witnessed an increasing concern of PS and related factors, particularly those associated with diseases. As researchers put more effort into investigating the PS properties of disease-related biomolecules, we decided that building a novel database concerning PS–disease associations would be pivotal, which leads to the development of PhaSeDis.

In the current version of PhaSeDis, we not only inherited the previous PS–disease association entries from MloDisDB but also collected all publications related to PS listed on National Center for Biotechnology Information (NCBI) PubMed by manually examining the search results of the keyword “(phase separation[Title/Abstract]) AND ((disease[Title/Abstract]) OR (cancer[Title/Abstract]) OR (neurodegeneration[Title/Abstract]))”. Considering that MloDisDB collected data up to April 2020, the time range was set as “2020–2022” to collect newer publications. All publications searched by previous keywords and filters were examined manually in full text for extracting comprehensive annotations. We only filtered publications with results showing *in vivo* evidence of factors (proteins or RNAs) phase separating into condensates or granules.

**Figure 1:**
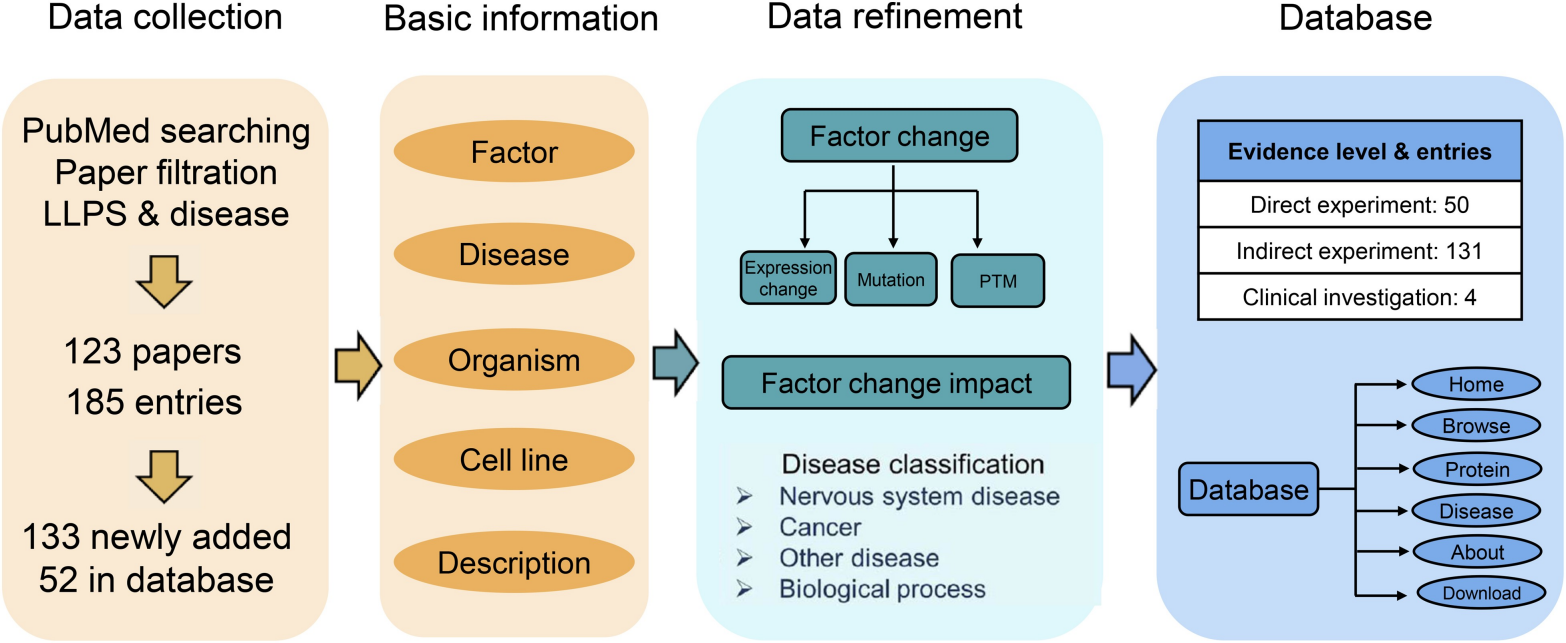
**Procedures of PhaSeDis data curation and construction**PhaSeDis data were derived from papers published between 2020 and 2022 retrieved from PubMed using the search term “(phase separation[Title/Abstract]) AND ((disease[Title/Abstract]) OR (cancer[Title/Abstract]) OR (neurodegeneration[Title/Abstract]))” and previous MloDisDB. We filtered 123 papers and curated 185 entries. These entries were annotated with phase separating factor name, disease, organism, cell line, and a brief description. Then we refined the data with details about expression changes, mutations, and PTM changes along with their impacts on pathological conditions, related disease classification, and evidence levels. All aforementioned basic information and data refinements were illustrated in the final database with a web interface. LLPS, liquid–liquid phase separation; PS, phase separation; PTM, post-translational modification.

As a result, we collected 553 publications from April 2020, the end point of MloDisDB, to December 2022. After going over all the literature, we curated 156 novel entries, with 133 being PS-related entries. That boosts the total number of PS-related entries from 52 to 185, and the total number of entries from 775 to 931. These 185 entries are derived from 123 papers, in which the authors provided direct experiment, indirect experiment, or clinical investigation evidence of PS-related factors affecting the corresponding diseases through PS. We followed the same protocol as in MloDisDB [27] in recording the properties of the phase separating factor [type, name, gene identifier (ID), and UniProt ID (if being a protein)], the information of related diseases [classification, name, disease ontology, Medical Subject Headings (MeSH), International Classification of Diseases 10 (ICD-10), Online Mendelian Inheritance in Man (OMIM), *etc.*], MLO changes, or the changes in phase separating droplets (size, number, assembly, dynamic, *etc.*), as well as the description in the paper indicating the mechanism of how the PS factor leads to the aforementioned diseases (**Figure 1**). In a nutshell, we doubled the PS–disease association entries in PhaSeDis, which reflects the advancement of recent PS research.

Next, we reviewed the 185 PS entries to assess whether the associated literature provides adequate evidence for the relationships between PS and diseases. Among the entries, 50 entries include direct PS-validating experiments, meaning that their results provide evidence that the biomolecules can influence cellular pathological conditions through PS; *e.g.*, MD788 indicates that glycogen PS is directly associated with hepatocellular carcinoma. As reported by Liu et al., when glycogen accumulates in liver cells and forms Laforin-mediated glycogen–Mst1/2 condensate, the Hippo signaling pathway will be turned off, leading to tumor initiation, which directly links PS with diseases [28]. Moreover, 131 entries indicate indirect proof, which includes evidence that a protein or RNA can lead to diseases and that the biomolecule can perform PS; *e.g.*, MD872 indicates an indirect association between androgen receptor (AR) PS and prostate cancer (PCa). Zhang et al. reported that AR-rich foci exhibit properties of liquid-like condensates and correlate with transcriptional activities in PCa cells, while the research didn’t provide results linking AR PS with tumor progression, making it an indirect association [29]. The other 4 records consist of clinical investigations; *e.g.*, the original publication of MD894 record includes human kidney samples in order to demonstrate that chromodomain Y like protein (CDYL) PS enhances its catalytic activity and attenuates cytogenesis through PS in an autosomal dominant polycystic kidney disease (ADPKD) model [30] (Figure 1). These results show that most research concerning PS still focuses on the phenomenon that proteins or other biomolecules condense as droplets.

**Figure 2:**
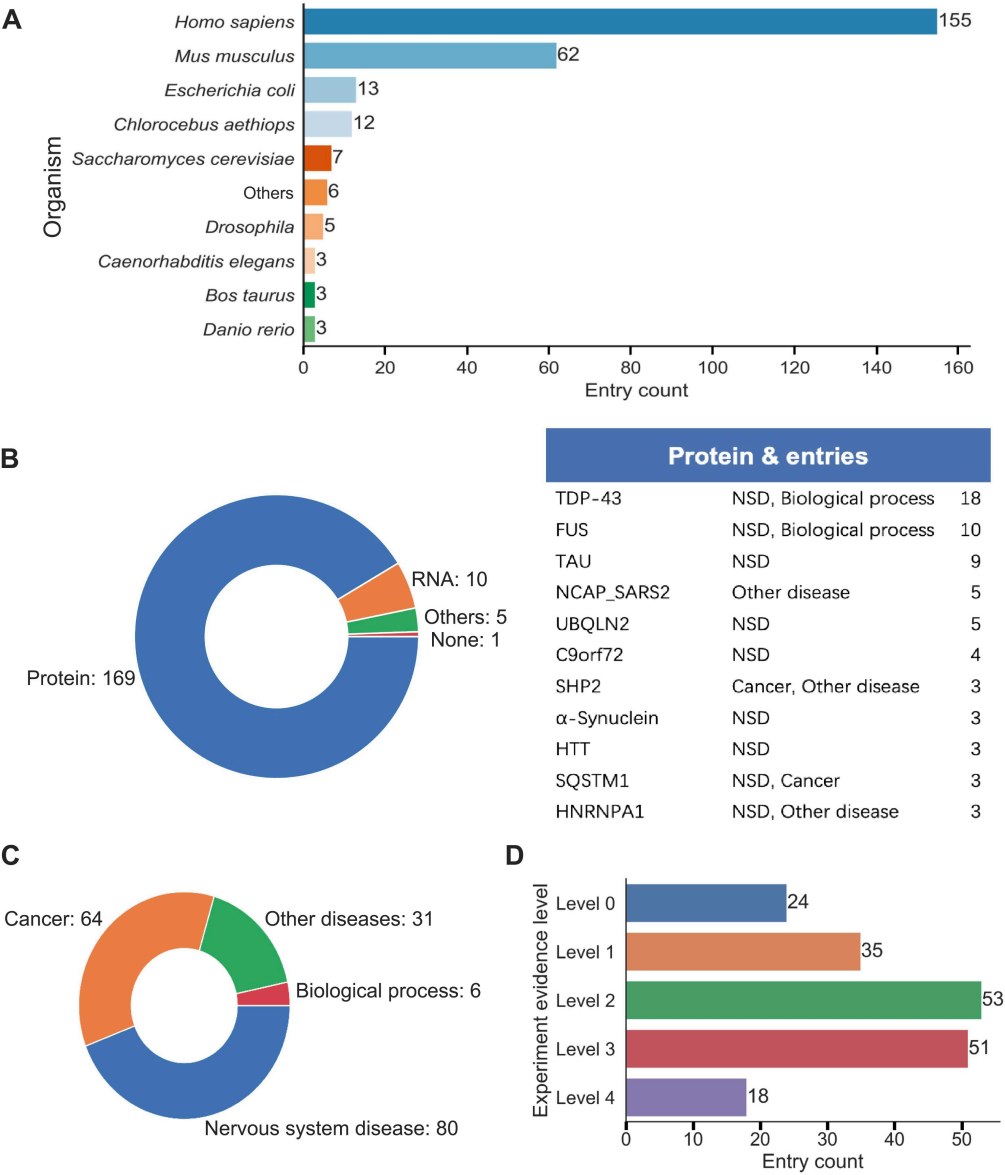
**Statistical analysis of entries in PhaSeDis** **A**. Distribution of the organisms of the entries. **B**. Left: distribution of the factor types of the entries. Right: a list of factors in PhaSeDis with the most appearances in the entries. **C**. Distribution of the diseases associated with the entries. **D**. Entry counts at different levels of PS evidence. Levels 0 to 4 stand for the number of valid *in vivo* experiments reported in the corresponding literature for each entry. NSD, nervous system disease.

As shown in **Figure 2**A, the majority of PS factors collected belong to *Homo sapiens*, almost doubling the number of PS factors in *Mus musculus*. Proteins still account for a large portion of all PS entries, while PS of RNAs also contributes to diseases (Figure 2B). Some key factors like TAR DNA-binding protein 43 (TDP-43) and fused in sarcoma (FUS) appear in more than 10 entries, highlighting the critical role of PS in these key factors concerning neurodegenerative diseases. We found that a wide variety of diseases are associated with PS, most of which are nervous system diseases and cancer (Figure 2C). To find out the specific diseases that PS has the most effects on, we also analyzed the distribution of PS factors in nervous system diseases and cancer, two of the largest categories where PS play a big part in. We found that among the 80 “nervous system disease” entries, 27 have relations to one rare neurodegenerative disease named amyotrophic lateral sclerosis (ALS), which is even greater than the number of related entries of a more general “neurodegenerative diseases” category (**Table 1**). On the cancer side, diseases are more evenly distributed than in the neurodegenerative disease sector. Lung non-small cell carcinoma has been the leading disease (8 entries), while the highest number of entries in the cancer category belongs to “cancer general process” (11 entries) (**Table 2**). These findings indicate that most cancer researchers tend to investigate the general mechanisms that PS plays in cancer. Besides, there are also a number of other diseases that PS has an influence on. For instance, there are 5 entries related to autosomal dominant polycystic kidney disease (ADPKD), 2 entries related to Noonan syndrome, a genetic disorder with heterogeneous phenotypic manifestations, *etc.* These results indicate that PS-related factors are widespread across many disorders and PS is a significant mechanism that should not be neglected in investigating these diseases.

**Table 1:** Number of PS entries related to nervous system diseases

Disease category	PS entry count
Amyotrophic lateral sclerosis	27
Neurodegenerative disease	20
Frontotemporal dementia	12
Alzheimer’s disease	8
Parkinson’s disease	3
Huntington’s disease	3

*Note*: PS, phase separation.

**Table 2:** Number of PS entries related to cancer

Disease category	PS entry count
Cancer general process	11
Lung non-small cell carcinoma	8
Breast cancer	5
Ewing sarcoma	4
Colorectal cancer	4
Hepatocellular carcinoma	3

One of the most concerning problems in the PS field is the verification of PS *in vivo*. In the biological context, as a novel concept, determining whether an MLO or granule forms via PS lacks a gold standard [12]. Therefore, we supplemented another novel property for each PS entry entitled “*in vivo* PS evidence”. We listed 4 of the most renowned experiments that can help to determine whether the protein can perform PS *in vivo*. (1) Fluorescence recovery after photobleaching (FRAP). This experiment labels PS factors with fluorescence, performs photobleaching on a small spot of area within the condensate with high-energy lasers, and records the fluorescence recovery time. FRAP allows for the comparison of viscosity between phase separating droplets. (2) 1,6-HD treatment. 1,6-HD is one of the most used small molecules that can dissolve PS droplets. A large amount of research would treat droplets with 1,6-HD to confirm their LLPS nature [14]. (3) Spherical morphology. The nature of LLPS indicates that the droplet tends to form a circular shape due to surface tension, since no membranes are bound to the droplet. (4) Fusion & fission phenomenon. When two droplets share similar viscosity and composition, they tend to fuse when their boundaries touch. A PS droplet can also divide into multiple small droplets. Considering the wide acceptance of these experiments when determining PS, we annotated the PS entries with the existence of said *in vivo* experiment evidence in the corresponding references. The sub-columns are annotated as “Yes” if the corresponding experiment was performed *in vivo* in the reference article mentioned in the entry, and “No” *vice versa*. Another sub-column named “PS role” indicates the possible role of the corresponding factor in forming the PS condensate, which could be “scaffold” if the molecule is the main condensing or recruiting factor, “client” if the molecule is being recruited or transported into the droplet, or “self” if the droplet is condensed by the molecule solely. After annotation we summed up the number of experiments that each entry has and named it as the “level of evidence” for the corresponding entry. Each entry ranges from “level 0” (having done none of the aforementioned validation experiments) to “level 4” (having done all validation experiments). As shown in Figure 2D, we can see that the majority of entries have a level 2 or level 3 of confidence. Only 18 entries have done all 4 experiments *in vivo* and 24 entries didn’t perform any *in vivo* PS property validations. These results indicate the possible levels of confidence that one can expect from a PS publication.

### Curation of related small molecules concerning PS

**Figure 3:**
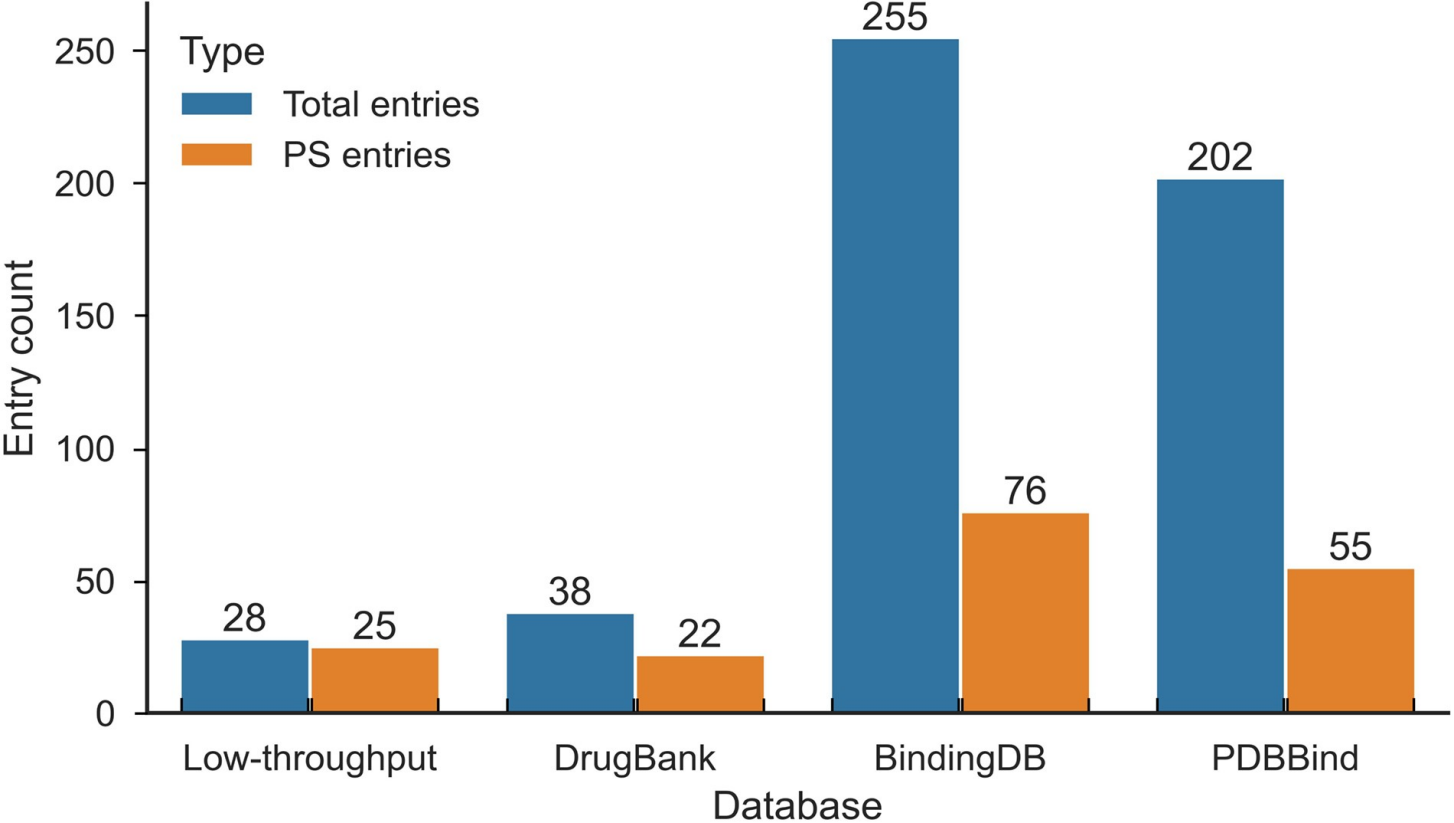
**Distribution of related small molecule data in PhaSeDis**The distribution of low-throughput evidence from corresponding literature is illustrated in the “low-throughput” column. Counts of high-throughput screening results searched against DrugBank, BindingDB, and PDBBind are shown in respective columns. BindingDB, Binding Database.

Previous studies have shown that small molecules can serve as drugs targeting disease-related proteins [31,32]. In PhaSeDis, we integrated these individual pieces of evidence and constructed a “Related small molecules” section for each entry. For each entry, we checked their corresponding reference publication in search of small molecules interacting with or disrupting PS and listed the description in “Interference note” of the “Related small molecules” section. We manually curated how small molecules influence the pathological processes from each reference publication and annotated them in the entry. Among all entries in the database, 28 entries include evidence that small molecules can regulate the pathological processes, and 25 of them are PS entries (**Figure 3**). This information provides low-throughput evidence of small molecule interference with PS *in vivo*.

Besides low-throughput experimental evidence from literature, many databases with information of small molecule–protein interaction data, including Binding Database (BindingDB), PDBBind, and DrugBank, would also include a wide range of small molecules and their targets [33–36]. Nevertheless, none of these databases would illustrate the significance of PS when it comes to small molecule targets. Therefore, based on our previous curation of PS protein–disease relations in PhaSeDis, we searched all PS proteins in our database against these databases. BindingDB provides a large amount of protein–small molecule interactions with experimental data. For BindingDB, we searched the UniProt IDs of all PS proteins in PhaSeDis and acquired a list of interacting small molecules for each protein. Since most of the molecules do not have a unified common name, we used their PubChem compound identifier (CID) as the unique representation of each molecule and annotated them to the corresponding entry as “Molecules from BindingDB”. We also searched our PS factor entries against PDBBind, a database with comprehensive information about protein–ligand and other interactions. Besides binding affinity data, PDBBind presents information about the structure details of protein–ligand binding, which increases the reliability of protein–ligand interactions. The small molecules derived from PDBBind were annotated to the corresponding entry as “Molecules from PDBBind”. Although having a higher tendency to interact with PS factors, molecules found in BindingDB and PDBBind do not necessarily qualify as potential drugs, since they do not provide information concerning diseases. Therefore, we also searched all PS proteins, both by gene ID and UniProt ID, in PhaSeDis against DrugBank, where most small molecules have greater probabilities to qualify as drugs for certain diseases. The interacting drugs related to each protein were annotated in the entry as “Drugs from DrugBank”. DrugBank serves as a database for drugs, indicating that small molecules from DrugBank targeting corresponding PS factors can affect diseases with pre-clinical or clinical evidence. In total, there are 255, 202, and 38 entries whose factors have relations with small molecules in BindingDB, PDBBind, and DrugBank, respectively. For verified PS entries, the number of entries with small molecule relations are 76, 55, and 22 for BindingDB, PDBBind, and DrugBank, respectively (**Figure 3**). These small molecules are listed in separate columns of the “Related small molecules” section and constitute the high-throughput evidence of possible PS-related small molecules. With these related small molecules, PhaSeDis provides an initial list of potential small molecules to start with when dealing with each individual PS protein.

## Database web interface

**Figure 4:**
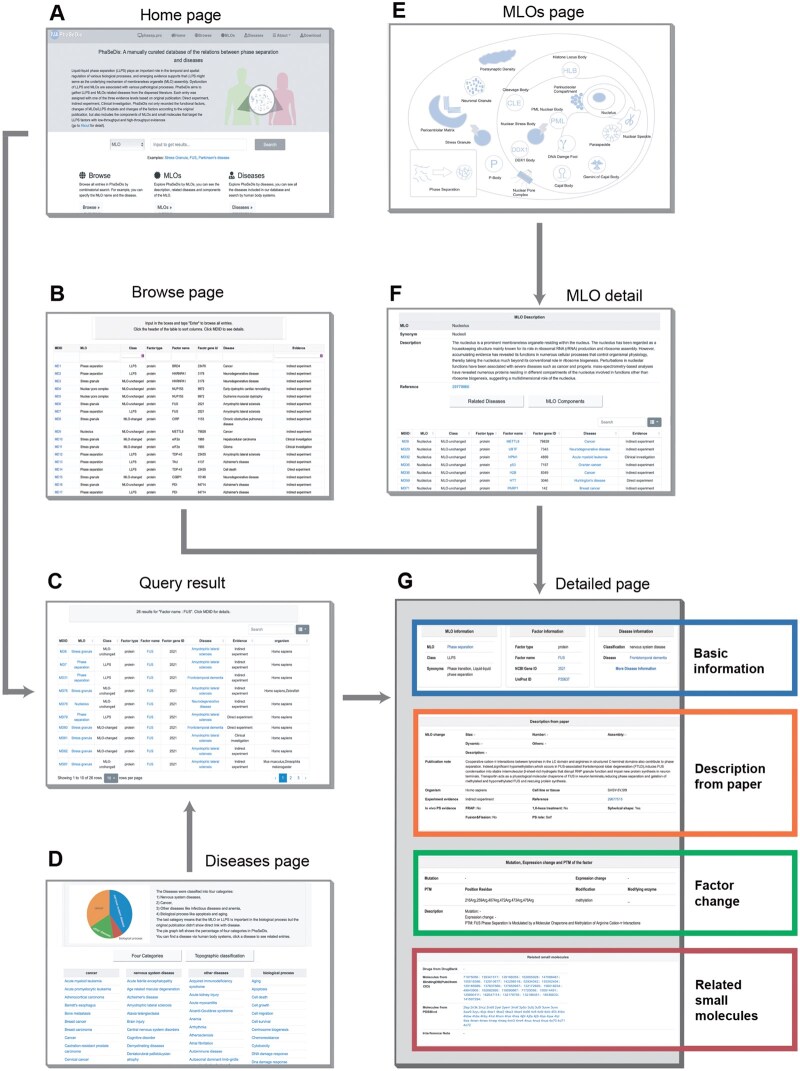
**The web interface of PhaSeDis** **A**. Home page. Users can search by MLO, factor name, factor gene identifier, or disease. **B**. Browse page. Users can browse entries of interest in a simple table form. **C**. Query result page. Users can examine query results upon searching and download them. **D**. Diseases page. Users can see all the diseases collected in PhaSeDis in classified tables. **E**. MLOs page. Users can navigate the MLOs through a graphical scheme. **F**. MLO detail page. Users can learn more about the MLO and its correlated entries. **G**. Detailed page. This page shows all information included in an entry. MLO, membraneless organelle.

The freely available and fully functional website of PhaSeDis (http://mlodis.phasep.pro) has been greatly improved in order to handle the updated information. The website contains six sections, namely “Home”, “Browse”, “MLOs”, “Diseases”, “About”, and “Download” (**Figure 4**A). Users can search entries of interest on the “Home” page by MLO, factor name, factor gene ID, disease, or organism, and download the search results as a tab-separated values (TSV) file or browse all entries on the “Browse” page. Users can also choose from all possible options from “Evidence” or “Class” types to browse one specific type of entries (Figure 4B). The query results are presented in a single table containing MLOs, factors, diseases, and entry groups (Figure 4C). The “Diseases” page provides a thorough list of all LLPS/MLO-related diseases illustrated in the database. Clicking on each disease name will bring users to all related entries in the database (Figure 4D). The “MLOs” page includes a straightforward scheme, providing a user-friendly graphical navigation enabling users to browse all entries related to an MLO of interest by simply clicking on the MLO (Figure 4E). There is also an “MLO detail” page providing a basic introduction to every MLO (Figure 4F). Users can click on “LLPS” on the “MLOs” page to see all LLPS–disease entries with confirmed LLPS proteins.

For every entry, we assigned a unique in-built ID named MDID, which can be seen on the “Browse” page, “Query result” page, or “MLO detail” page. Clicking on any MDID would bring users to the detailed page of the entry (Figure 4G). The detailed page of each entry consists of four parts. (1) Basic information, which contains the information of MLO, factor, and disease. (2) Description from paper, which encompasses the changes in MLO or LLPS bodies in terms of size, number, assembly conditions, dynamic, and others, as well as a “Publication note” introducing how condensation might participate in pathological processes. This section also includes the PubMed ID of the original article, as well as the organism, cell/tissue type, and experimental evidence. For LLPS-related entries, the dataset includes annotations indicating whether *in vivo* evidence for PS is present, as well as the functional role of the corresponding factor in PS processes (classified as self, scaffold, or client). (3) Factor change, which includes all mutations, expression changes, and post-translational modification (PTM) changes reported in the original article and their impact. (4) Related small molecules, which illustrates both low-throughput and high-throughput evidence of targeting or interacting small molecules. The low-throughput evidence regarding the impact of related small molecules on cellular or organismal pathological conditions is collected from literature sources and detailed in the “Interference notes” section. These indicate the potential changes observed when the small molecules are introduced. Conversely, small molecules supported by high-throughput evidence are systematically linked to their corresponding databases.

User guide and data summary are detailed on the “About” page. On the “Download” page, we added a “PhaSeDis data” item, with the description: “This table contains all entries in PhaSeDis, including information about related small molecules.” This allows users to freely download all data in PhaSeDis for further analysis.

## Discussion

PS can help explain the concentration and sequestration of certain proteins or nucleic acids, which can accelerate or inhibit certain biological functions and promote the formation of MLOs. Dysregulation of PS is an important pathological mechanism, particularly in nervous system diseases and cancer. Previous studies have identified data resources linking condensate dysregulation with potentially contributing genetic variations in putative condensate-forming proteins [37]. However, due to a lack of “golden standard”, researchers do not have a consensus of the detrimental experimental observation and illustrate the PS properties of proteins with different sets of methodologies. In this work, we updated the PS entries in PhaSeDis to include the precise experimental evidence of the factor in the original publications, allowing a more comprehensive view on the reliability of each entry. We have exclusively included *in vivo* PS evidence because such data are more reflective of actual physiological conditions within a biological context compared to *in vitro* experiments.

Besides, PS properties of biomolecules pose new challenges to drug discovery, since small molecules might have to partition into or dissolve condensates to affect diseases [8]. A large body of literature has issued the influence of small molecule treatments on diseased cells or animal models [16,20,38]. In PhaSeDis, we collected both low-throughput evidence from literature and high-throughput evidence from protein–small molecule interaction databases including BindingDB, PDBBind, and DrugBank. These databases provide possible small molecule interaction data from different aspects: BindingDB focuses on the binding nature of small molecules with PS factors related to diseases; PDBBind includes a variety of small molecules/ligands binding to proteins at specific regions, which can be displayed as 3-dimensional (3D) structures and stored in Protein Data Bank (PDB) files; DrugBank provides a list of approved small molecules that are more likely to deal with the disease from the respective entry. We do not intend to imply that these small molecules necessarily bind to or interfere with PS factors through any specific mechanisms, nor do we suggest that these molecules themselves undergo PS. In fact, research suggests that certain small molecules might not operate in the conventional manner of drugs that bind to target pockets; rather, they could potentially exert their disease-modifying effects through noncanonical modes of action [39]. It should be noted that BindingDB, PDBBind, and DrugBank are not specifically focused on PS. Nevertheless, PhaSeDis has already linked disease-related proteins with PS, as indicated in the “Description from paper” section of each record. These databases provide high-throughput evidence for the connection between small molecules and proteins, which is integrated into PhaSeDis. While these molecules are presumed to be related to PS, we have not indicated that they necessarily regulate or interfere with PS through any specific mechanisms. However, these small molecules, derived from protein–small molecule interaction databases, have a higher probability of targeting key PS factors. Besides small molecule–protein interaction databases, there are also databases that introduce the relations between small molecule perturbance and gene expression changes in a variety of diseases, *e.g.*, Connectivity Map (cMAP) [40]. Nevertheless, the relationship between gene expression changes and protein binding is complex and not straightforward to establish. Therefore, as a database specifically dedicated to the relationship between PS (proteins/RNAs) and diseases, PhaSeDis does not incorporate data from cMAP.

In recent literature, there are also other databases that focus on the relationship between PS and diseases, including DisPhaseDB [41]. Although PhaSeDis and DisPhaseDB both include information about PS protein–disease relations, there are some differences between the two databases. DisPhaseDB collects proteins with LLPS evidence from databases and collects related diseases based on the mutations in ClinVar, with a focus on “variants”. PhaSeDis collects PS factors and their relations to diseases in existing literature, as well as potentially interfering small molecules from literature and databases. As shown in Figure S1, there are 231 proteins overlapping between the two databases; however, PhaSeDis contributes additional 117 unique proteins that are not covered in DisPhaseDB, accounting for roughly one third of all PhaSeDis entries (Figure S1). PhaSeDis is also a great supplement to existing PS factor databases, *e.g.*, PhaSePro [24]. As shown in Figure S2, PhaSeDis and PhaSePro have only 25 shared proteins, with 323 unique in PhaSeDis. PhaSeDis incorporates more recent research findings and is specifically dedicated to diseases where PS factors play a role and how small molecules can intervene in these processes, taking a distinct approach compared to other PS databases that primarily concentrate on cataloging LLPS proteins.

Currently, systematically summarizing mechanisms of diseases involving PS, as well as mechanisms of small molecules targeting PS, remains challenging and cannot rely on any single property [42,43]. Researchers dedicated to different diseases have their own hypotheses about how PS affects disease progression and alleviation [10,44]. For PS-intervening small molecules, some researchers have put forward a few classifications of mechanisms [45], and our data can partially fit in: (1) some molecules work as dissolvers, like in MD787, where phospholipase D inhibitor 1-butanol or 2-butanol decreases large tumor suppressor kinase 1 (LATS1) puncta numbers [15]; (2) some molecules work as inducers, like in MD792, where sulforaphane (SFN) treatment doubles the number of super-enhancers to increase nuclear factor erythroid 2-related factor 2 (NRF2) activity and improve kidney function for ADPKD patients [16]; (3) some molecules work as localizers, like in MD838, where 5-fluorouridine and ebselen target superoxide dismutase 1 (SOD1) at W32S and C111S sites to prevent oxidation-induced aggregation [17]; (4) some molecules work as morphers, like in MD851, where lipid incorporation into α-synuclein droplets delays their maturation process, thereby rendering the droplets more resistant to aging [18]. However, other types of mechanisms have also been proposed [9]. For instance, it could also stem from the conformational changes induced by drug binding, which effectively interfere with and hinder the progression of pathological conditions [46], as in MD814–MD816, where Src homology region 2 domain-containing phosphatase-2 (SHP2) allosteric inhibitor attenuates LLPS of SHP2 mutants [32]. As the currently available evidence does not permit us to draw definitive conclusions about the specific nature of drug–PS protein–disease interactions, PhaSeDis aims to provide detailed introduction to every entry concerning how PS factors lead to pathological conditions through PS and how small molecules intervene. As PhaSeDis accumulates more data, we hope that it will facilitate deeper understanding of the *in vivo* roles of PS phenomena, their associations with diseases, and the identification of potential therapeutic agents. For now, the current version of PhaSeDis serves as a valuable starting point for initiating a multitude of research inquiries into drug–PS protein–disease interactions.

There are also a few limitations on this work. Firstly, the number of PS factors is limited, which makes it difficult to summarize and categorize the features of the PS-related pathological mechanisms. Secondly, the protein–small molecule databases from which we source our small molecules do not specifically emphasize PS aspects. Therefore, researchers need to further investigate and validate the mechanisms by which these small molecules potentially influence PS. Besides, the connection between protein/RNA-induced disease and phase-separating biomolecules may be tentative based on preliminary evidence in the literature. As the development of relevant research and continuous update of annotations, PhaSeDis would become more and more informative.

Taken together, PhaSeDis has grown into a comprehensive database for PS factor–disease relations, as well as the first database of its kind that integrates small molecule interaction in the database. These invaluable resources will greatly assist researchers who have a keen interest in the realm of PS, empowering them not only to rigorously substantiate the intricate partitioning mechanisms of currently available medications but also to embark on pioneering quests for novel therapeutic agents capable of effectively disrupting PS. Such endeavors hold immense promise for advancing our understanding of PS, ultimately contributing to the development of innovative treatment strategies.

## Supplementary Material

qzaf014_Supplementary_Data

## Data Availability

PhaSeDis is publicly available at http://mlodis.phasep.pro. As an updated version of MloDisDB, detailed information about this database has been submitted to Database Commons [47] at the National Genomics Data Center (NGDC), China National Center for Bioinformation (CNCB), which is publicly accessible at https://ngdc.cncb.ac.cn/databasecommons/database/id/7148.

## CRediT author statement

**Taoyu Chen:** Conceptualization, Methodology, Formal analysis, Investigation, Data curation, Writing – original draft. **Guoguo Tang:** Formal analysis, Investigation, Data curation, Writing – review & editing, Visualization. **Tianhao Li:** Methodology, Software, Investigation, Formal analysis, Visualization. **Zhining Yanghong:** Software, Formal analysis, Visualization. **Chao Hou:** Software. **Zezhou Du:** Data curation. **Kaiqiang You:** Writing – review & editing. **Liwei Ma:** Writing – review & editing. **Tingting Li:** Conceptualization, Methodology, Writing – review & editing, Supervision, Project administration, Funding acquisition. All authors have read and approved the final manuscript.
