# Supplementary material for: PhaSeDis: A Manually Curated Database of Phase Separation–disease Associations and Corresponding Small Molecules
Source: Genomics Proteomics Bioinformatics. 2025 Mar 4;23(1):qzaf014. doi: 10.1093/gpbjnl/qzaf014 (PMC12208530; doi:10.1093/gpbjnl/qzaf014)
Supplement: qzaf014_Supplementary_Data [file qzaf014_supplementary_data.zip › supplementary_material_captions.docx]

**Supplementary material**

**Figure S1 Venn diagram of the distinction between the proteins contained within DisPhaseDB and PhaSeDis databases**

There are 231 proteins overlapping between the two databases, while 117 proteins are unique to PhaSeDis and 5510 proteins are unique to DisPhaseDB.

**Figure S2 Venn diagram of the distinction between the proteins contained within PhaSePro and PhaSeDis databases**

There are 25 proteins overlapping between the two databases, while 323 proteins are unique to PhaSeDis and 96 proteins are unique to PhaSePro.
