# Supplementary figures and images for: PhaSeDis: A Manually Curated Database of Phase Separation–disease Associations and Corresponding Small Molecules
Source: Genomics Proteomics Bioinformatics. 2025 Mar 4;23(1):qzaf014. doi: 10.1093/gpbjnl/qzaf014 (PMC12208530; doi:10.1093/gpbjnl/qzaf014)

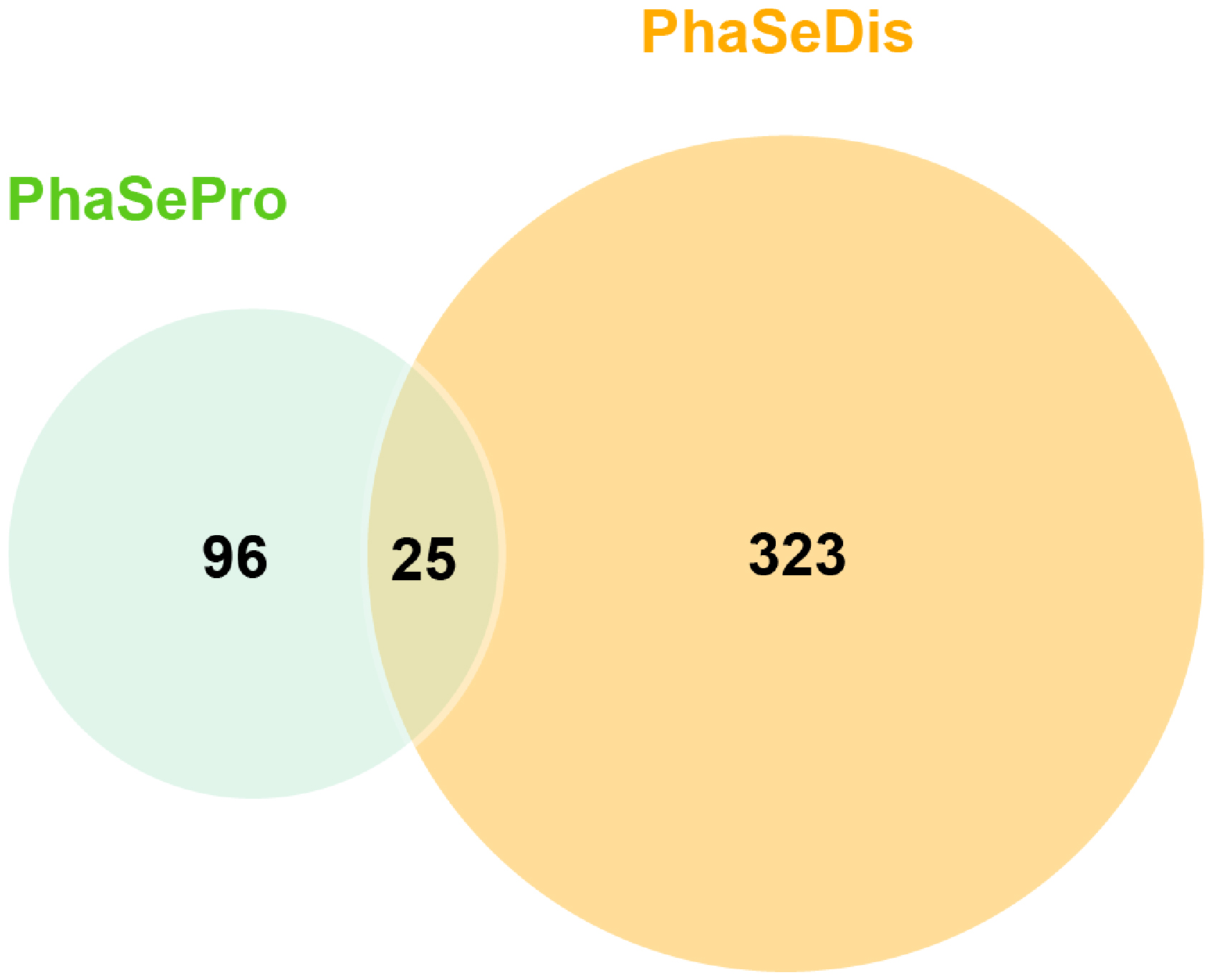

Supplement: qzaf014_Supplementary_Data [file qzaf014_supplementary_data.zip › FigS2.jpg]

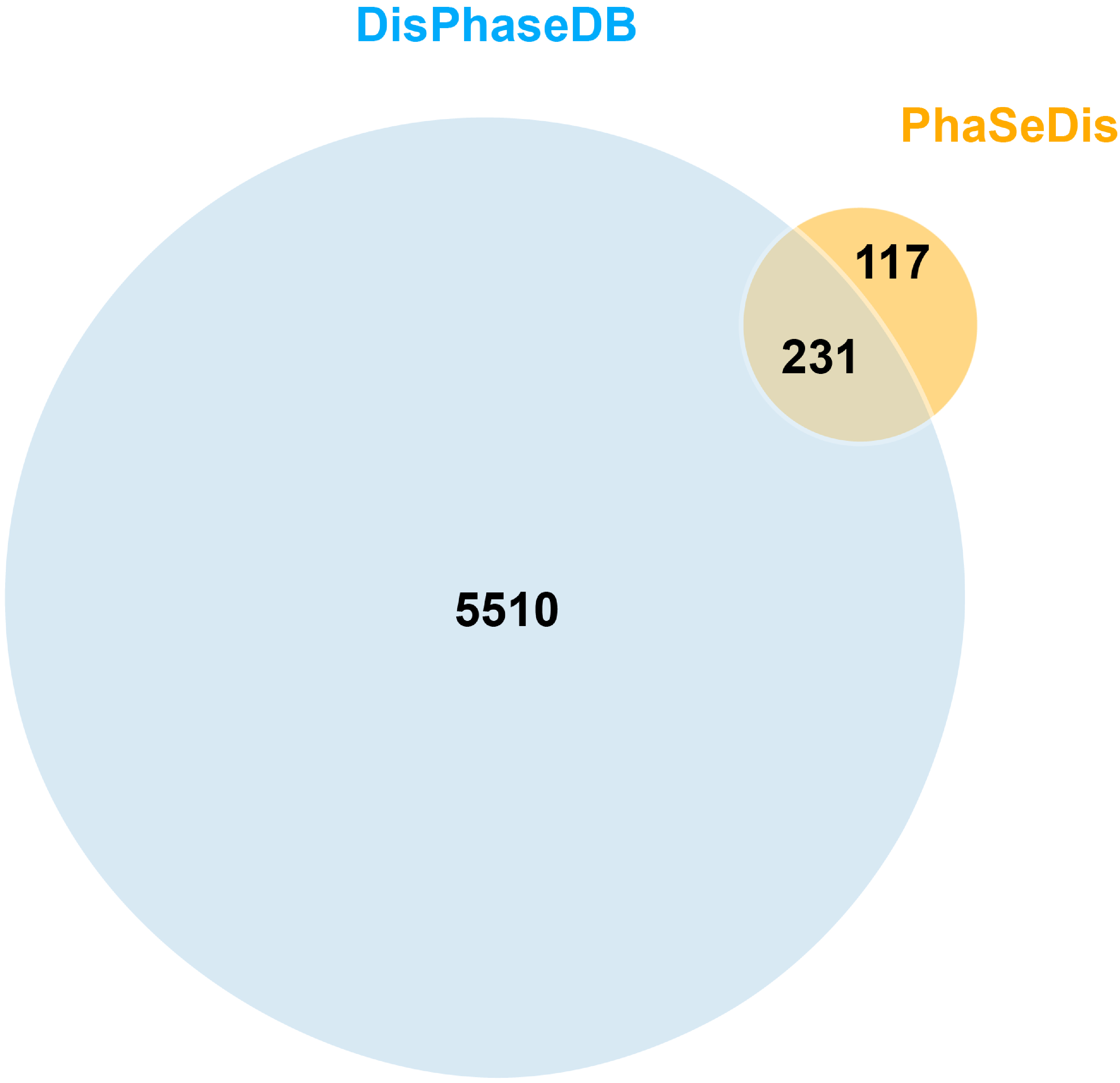

Supplement: qzaf014_Supplementary_Data [file qzaf014_supplementary_data.zip › FigS1.jpg]
